# Fbxo21 regulates the epithelial-to-mesenchymal transition through ubiquitination of Nr2f2 in gastric cancer

**DOI:** 10.7150/jca.49674

**Published:** 2021-01-01

**Authors:** Yannan Jiang, Xinyu Liu, Renbin Shen, Xinhua Gu, Weifeng Qian

**Affiliations:** 1 Department of General Surgery, The Affiliated Suzhou Hospital of Nanjing Medical University.; 2 Department of General Surgery, The First Affiliated Hospital of Zhengzhou University.

**Keywords:** Fbxo21, gastric cancer, Nr2f2, proliferation, EMT

## Abstract

F-box protein 21 (Fbxo21), a member of the F-box family proteins, constitutes one of the four subunits of an E3 ubiquitin ligase complex called SCFs (SKP1-Cullin-F-box). Despite the effect on antivirus immune response and ubiquitination regulation of a few oncoproteins, such as EID1 and P-gp, little is known about the Fbxo21 function in tumors, including gastric cancer. In our study, we confirmed that Fbxo21 expression was decreased in gastric cancer tissues. Decreased expression of Fbxo21 was significantly associated with poor prognosis in gastric cancer. Fbxo21 inhibited gastric cancer progression by inducing growth arrest and inhibiting migration and invasion. The expression of various EMT markers, such as E-cadherin, N-cadherin and Vimentin were altered after Fbxo21 knockdown or overexpression. Moreover, we demonstrated that Fbxo21 inhibited the EMT via the down-regulation of Nr2f2. Fbxo21 expression was negatively correlated with Nr2f2 protein expression in gastric cancer tissues and cell lines. And the Nr2f2 protein abundance was regulated by Fbxo21 via ubiquitination and proteasomal degradation. At last, we demonstrated the effects of Nr2f2 re-expression and inhibition on stable Fbxo21-overexpression or Fbxo21-silenced cell lines. These results suggested that Fbxo21 inhibited the proliferation and EMT in part through down-regulating the Nr2f2.

## 1. Introduction

Gastric cancer is one of the most common malignant tumors with high mortality 1. It accounts for the second place in incidence and the third in cancer-related mortality in China 2. Lots of patients are diagnosed with advanced gastric cancer initially, and the surgery only is unable to cure. About half of the patients would have post-operative recurrence or metastasis, with an average survival of less than 12 months 3. Gastric cancer invasion and metastasis is a biological process involving multiple factors and steps. Epithelial-to-mesenchymal transition (EMT) is the key one 4. The epithelial cell loses its polarity and intercellular adhesion, and acquires a mesenchymal phenotype 5. Five signaling pathways were reported to be the main molecular mechanism of tumor EMT process, including Notch, Wnt/β-catenin, NF-κB, STAT3 and TGF-β pathway 6-8. The activation of these pathways is usually accompanied with loss or enhancement of EMT markers, such as Snail, Vimentin, E-cadherin and N-cadherin. Therefore, the molecular mechanisms that regulate gastric cancer cell invasion and migration behavior, especially the EMT progress should be further explored for the development of new therapies.

Fbxo21 (F-box only 21), a new-found member of the F-box protein family, is one of components of the SKP1-CUL1-F-box protein (SCF) E3 ubiquitin ligase complex, which can recognize and bind to target proteins, leading to their ubiquitination and proteasomal degradation 9. F-box proteins have been shown to anticipate in the gastric cancer development and regulate the EMT process, through targeting and degradation of specific EMT transcription factors directly, or controlling the EMT inducers, such as Notch, c-Myc or mTOR 10-13. For example, the widely studied tumor suppressor Fbxw7 induced the Snail ubiquitination and degradation in non-small cell lung cancer 14, controlled the RhoA protein level and the downstream Snail or Zeb1 expression in gastric cancer 15. Previous studies on Fbxo21 mainly focused on the immune response 16.However, the role in tumor has not been clearly reported. Only a few oncoproteins have been identified to be the targets of Fbxo21 for degradation, such as EID1 and P-gp 17-19. Therefore, considering the important role of F-box family proteins in tumor progression, we are interested in the Fbxo21 expression level and function in gastric cancer.

Nr2f2 (nuclear receptor subfamily2, group F, and member 2), also known as COUP-TF II, encodes a steroid/thyroid hormone receptor superfamily protein, and plays an important role in tumor metastasis, especially in EMT transformation 20, 21. Nr2f2 functions through several downstream proteins in EMT main signaling pathways, such as Snail, FOXM1, Smad4 and Smad7 22-26. Meanwhile, Nr2f2 was regulated by some upstream microRNAs, such as miR-382 and miR-27b 27, 28. These connections constitute a whole signaling pathway network affecting tumor migration and invasion by regulating EMT process. Nr2f2 usually functions through binding to 5'-AGGTCA-3' repeats directly, or co-reacting with other transcription factors 29. In gastric cancer, Nr2f2 was highly expressed in cancer tissues and cells, and related to poor prognosis and metastasis 28.

In this study, we demonstrated that Fbxo21 acted as a tumor suppressor by inhibiting proliferation and anti-EMT activities. Its expression was inversely correlated with Nr2f2 protein expression in gastric cancer, and Fbxo21 regulated Nr2f2 status by inducing Nr2f2 ubiquitination and proteasomal degradation. Importantly, we further showed that Fbxo21 regulated proliferation and the EMT process through the Nr2f2/Snail pathway.

## 2. Materials and methods

### 2.1. Tissue samples and immunohistochemistry

Gastric cancer samples and paired normal mucosa samples were obtained from the The Affiliated Suzhou Hospital of Nanjing Medical University between 2017 and 2018. None of the patients had received chemotherapy before the operation. The samples were immediately stored in RNAlater at -80℃ and formaldehyde at room temperature after collection. Immunohistochemistry were performed as described using antibodies against Fbxo21 (1:200 dilution, Abcam, USA) and Nr2f2 (1:50 dilution, Sangon Biotech, China). This study was approved by the Medical Ethics Committee of the The Affiliated Suzhou Hospital of Nanjing Medical University.

### 2.2. Cell culture and transfection

The SGC-7901, BGC-823, MGC-803, MKN-45, MKN-28 and AGS human gastric cancer cell lines, the GES-1 human gastric mucosa epithelial cell line were purchased from the cell resources of the Shanghai Institute of Digestive Surgery. The cell lines were cultured in DMEM (Hyclone, China) with 10% NBS (Every Green, China) and 1% penicillin/streptomycin (Sangon Biotech, China) in these media. All cells were cultured in an incubator containing with 95% air and 5% CO_2_ at 37℃. The Fbxo21-siRNA, Flag-Fbxo21, Nr2f2-siRNA, HA-Nr2f2 and the negative control vector were purchased from Genepharma Co. (Shanghai, China). The Fbxo21-siRNA target sequence: si1, GCCTTCCCTTATGAAACACTA, si2, CCTGGACATCTTTGACTACAT, si3, CGGCAACAGAAGATCTTAAAT. The Nr2f2-siRNA target sequence: TGCAAGCGGTTTGGGACCTTGAACA.

### 2.3. CCK8 and Colony formation assays

The cells were resuspended and seeded in 96-well plates in triplicate at a density of 1×10^3^ cells per well in 100μl of DMEM. In the following week, we assessed cell numbers using the Cell Counting Kit-8 (CCK8) assay (Beyotime, China) daily. For colony formation assay, the resuspended cells were seeded in 6-well plates in triplicate at a density of 800 cells per well in 2ml of DMEM and cultured for 2 weeks. Finally, the cells were stained with crystal violet, pictured and counted.

### 2.4. Transwell migration and invasion and wound healing assays

The cells were resuspended and added to the top chamber of a transwell insert (Millipore, USA) at a concentration of 1×10^5^ cells in the serum-free DMEM medium, and medium supplemented with 10% NBS was added to the bottom chamber. After 24h, the cells were fixed with 4% paraformaldehyde for 30min and then stained for 10 min in 0.1% crystal violet, pictured and counted. For invasion assay, the inserts were coated with Matrigel (BD Bioscience, USA) before adding the cells. Six visual fields were chosen to calculate the number of migrated cells. For the wound healing assay, the cells were cultured in 6-well plates and scratched with a 20μl tip when the plate was filled. Cells were observed at 0, 24 and 48h after the scratches were made.

### 2.5. Co-immunoprecipitation (co-IP)

We obtain the total protein lysates using RIPA cell lysis buffer first, then added 1μg of primary antibody into total protein lysates, and shook the mixture on a rotating shaker at 4℃, overnight. The next day, we added the protein A+G beads (Beyotime, China) to the mixture and rotated sample at 4℃ for 1h. The sediments were separated using instantaneous high-speed centrifugation, washed three times with PBS, and resuspended with 1× sample loading buffer. Then the agarose beads-antigen-antibody complexes were boiled for 10 min and used for western blot assays subsequently.

### 2.6. RT-PCR

We obtain the total RNA using RNA Purification Kit (Sangon Biotech, China) and assess the concentration and purity of the total RNA samples in a UV spectrophotometer. The primers for Fbxo21, Nr2f2 and Gapdh were as follows: Fbxo21 (forward 5'-CACGTTCCTTGTAATGGCTTCA-3', reverse 5'-ACGACTCATAGTCATCTGGCTG)-3'; Nr2f2 (forward 5'-TCATGGGTATCGAGAACATTTGC-3', reverse 5'-TTCAACACAAACAGCTCGCTC-3'); Gapdh (forward 5'-GGAGCGAGATCCCTCCAAAAT-3', reverse 5'-GGCTGTTGTC-ATACTTCTCATGG-3'). All experimental procedures were performed according to the instructions in the RT-PCR Kit (Sangon Biotech, China).

### 2.7. Western blot

The cells were incubated in RIPA cell lysis buffer (Solibare, USA) with phosphatase and protease inhibitor Cocktail (Selleck, China). The concentration of protein was quatified using a bicinchoninic acid protein assay kit. A total of 30μg of protein was resolved via SDS-PAGE and transferred to PVDF membranes. The membranes were blocked with 5% non-fat dry milk in TBST for 2h and incubated with the corresponding primary antibodies at 4℃, overnight. The antibodies were Fbxo21 (1:1000 dilution, Abcam, USA); Nr2f2 (1:1000 dilution, Sangon, China); E-cadherin (1:1000 dilution, CST, USA); N-cadherin (1:1000 dilution, CST, USA); Vimentin (1:1000 dilution, CST, USA); Snail (1:1000 dilution, CST, USA); Zeb1 (1:1000 dilution, CST, USA); ubiquitin (1:1000 dilution, Beyotime, China) and Gapdh (1:1000 dilution, Beyotime, China). After the incubation with secondary antibodies for 2h, membranes were visualized with an enhanced chemiluminescence kit (Beyotime, China) using a Tanon system (Tanon, China).

### 2.8. Nude mouse tumor transplantation model

All animal experiments were performed with approval of the Local Medical Experimental Animal Care Commission of The Affiliated Suzhou Hospital of Nanjing Medical University. Four-week-old male nude BALB/C mice were injected with SGC-7901-shRNA or control cells. Tumor length and width were measured every three days. All the mice were sacrificed after a month. For lung metastasis experiments, 2ⅹ10^6^ cells were injected into caudal vena. All the mice were sacrificed after 2 months. The pulmonary metastatic tissues and transplanted tumors were removed and fixed with 10% formalin for subsequent studies.

### 2.9. Statistical analysis

All experimental results were repeated at least three times and presented as mean and standard errors. Single comparisons between two groups were evaluated via Student's t-test using SPSS 20.0. The association between Fbxo21 expression and Nr2f2 expression was determined using the Spearman's rank correlation analysis. P<0.05 was considered statistically significant.

## 3. Results

### 3.1. Fbxo21 expression is reduced in gastric cancer and indicates a poor prognosis

**Figure 1:**
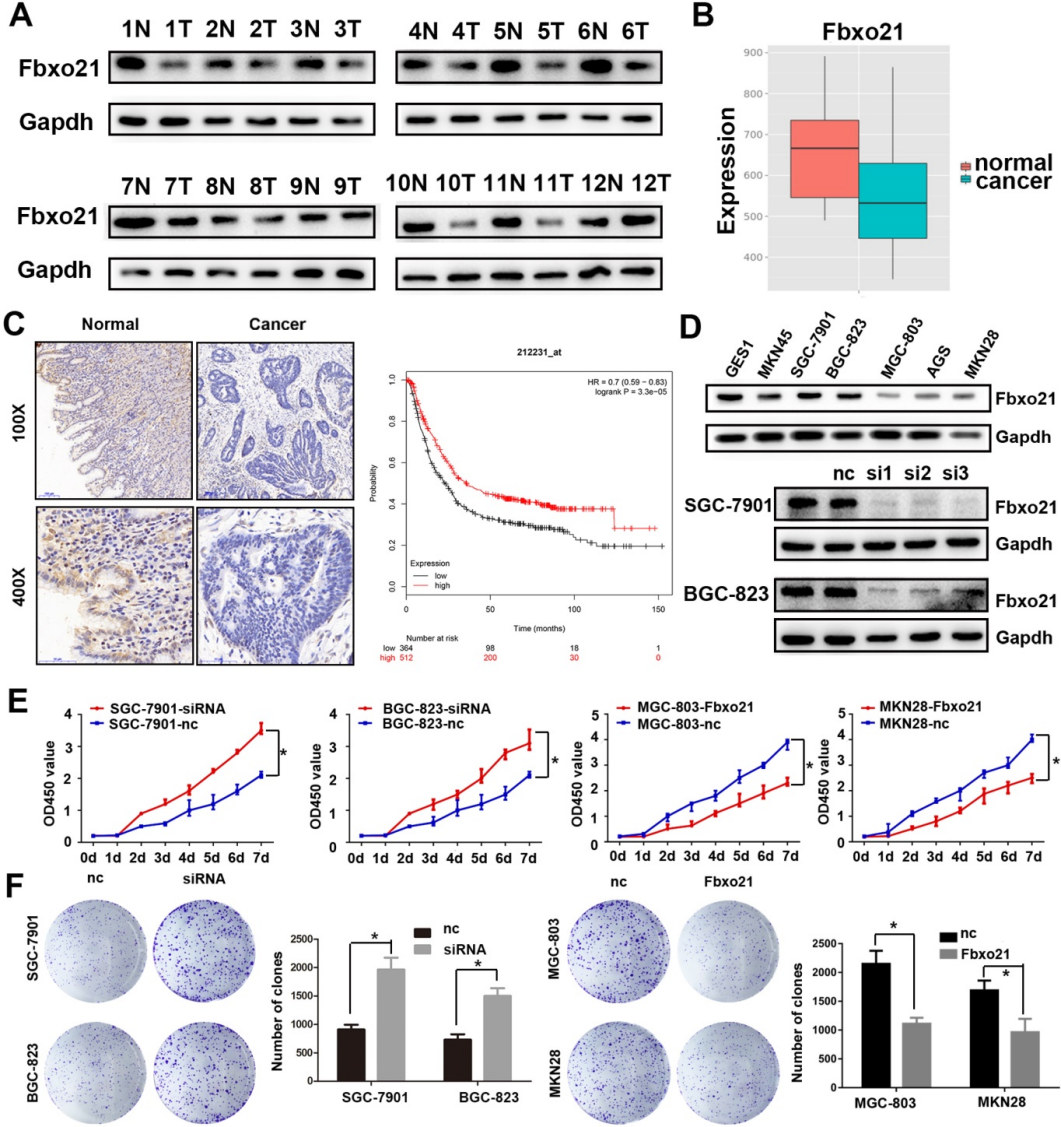
**Fbxo21 expression and its critical significance in gastric cancer patients. A.** Western Blot of Fbxo21 expression in matched normal tissues and gastric cancer tissues. **B.** Fbxo21 mRNA expression in normal and gastric cancer tissues from MERAV. **C.** Immunohistochemical (IHC) staining for Fbxo21 in normal and gastric cancer tissues. The results revealed that Fbxo21 expression in gastric cancer tissues was significantly lower than that in normal tissues. Kaplan-Meier 5-year overall survival curves for gastric cancer patients according to their Fbxo21 mRNA expression status from TCGA database. Significant differences in the overall survival rate are indicated (P<0.05). **D.** Fbxo21 expression in various gastric epithelial cell lines. The data showed that Fbxo21 expression was lower in the MKN45, SGC-7901, BGC-823, MGC-803, AGC, MKN28 than in the GES-1 cells. B. SiRNA used to knockdown the Fbxo21 expression in SGC-7901 and BGC-823 cell lines. The siRNA-1 had the best interference efficiency. **E.** The cell proliferation rate was increased in the Fbxo21-silenced SGC7901 and BGC-823 cells and decreased in Fbxo21-overexpression MGC-803 and MKN28 cells compared with the negative control cells based on CCK8 assay (*P<0.05). **F.** The colony formation assay showed the similar results (*P<0.05).

We detected Fbxo21 expression in 16 gastric cancer specimens and normal mucosa tissues after surgery using western blot and immunohistochemistry. The Fbxo21 protein level in gastric cancer tissues was significantly lower than that in matched normal tissues (Figure 1A and 1C). The Fbxo21 mRNA expression data obtained from MERAV website showed a similar result (Figure 1B) (http://merav.wi.mit.edu/SearchByGenes.html). Then we performed Kaplan-Meier survival analysis using overall survival data from TCGA. The results showed that the overall survival rate in Fbxo21-high group was significantly better than that in Fbxo21-low group (P<0.01) (Figure 1D). These data indicated that Fbxo21 expression is reduced in gastric cancer and correlates with a poor prognosis.

### 3.2. Fbxo21 inhibits cell proliferation in gastric cancer

As shown above, Fbxo21 expression level in gastric cancer is much lower than that in normal mucosa tissues. We also examined Fbxo21 protein level in seven cell lines: GES1, MKN45, SGC-7901, BGC-823, MGC-803, AGS and MKN28 cells, and found a similar trend to the results in tissue: Fbxo21 protein expression was lower in six gastric cancer cell lines (Figure 1D), compared with GES1. Thus, we hypothesized that Fbxo21 could play a significant role as a tumor suppressor in gastric cancer. To confirm the role of Fbxo21 in proliferation of gastric cancer cells, we transfected Fbxo21 siRNA into SGC-7901 and BGC-823 cells, and Flag-tagged Fbxo21 expression construct into MGC-803 and MKN28 dells (Figure 1E). Using CCK8 assay and colony formation assay, we found that Fbxo21 knockdown promoted cell proliferation in SGC-7901 and BGC-823 cells, while the Fbxo21 overexpression prevented the proliferation in MGC-803 and MKN28 dells (Figure 1F). Thus, our data confirmed that Fbxo21 could inhibit the cell proliferation in gastric cancer.

### 3.3. Fbxo21 regulates gastric cancer migration and invasion

**Figure 2:**
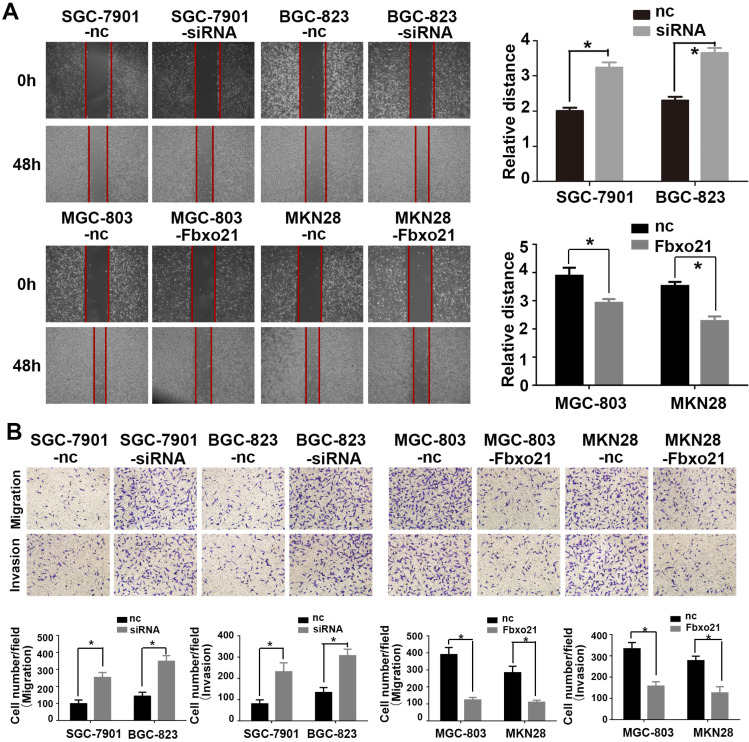
** Effects of Fbxo21 knockdown and overexpression on cell invasion and migration. A.** Wound healing assay of the Fbxo21-silenced SGC-7901 and BGC-823 cells and Fbxo21-overexpression MGC-803, MKN28 cells. **B.** The transwell assay of the Fbxo21-silenced SGC7901 and BGC-823 cells and Fbxo21-overexpression MGC-803, MKN28 cells. The migration and invasion capacities changed in these cells significantly, compared with negative control cells (*P<0.05).

The ability of migration and invasion of cancer cells is very important for the metastasis of gastric cancer. Therefore, in order to prove whether Fbxo21 is involved in the regulation of these processes of gastric cancer, we used SGC-7901, BGC-823, MGC-803 and MKN28 cells that were transduced with the Fbxo21-siRNA vector, the Fbxo21 expression vector or the negative control vector, and assessed cell migration and invasion using the transwell migration assay, the wound healing assay and the Matrigel-coated transwell assay, respectively (Figure 2). The results showed that the migration and invasion of SGC-7901 and BGC-823 cells in the Fbxo21 knockdown group were significantly increased compared with the negative control group. In parallel, MGC-803 and MKN28 cells overexpressing Fbxo21 had lower migration and invasion capacities. In conclusion, Fbxo21 regulated the migration and invasion of gastric cancer cells.

### 3.4. Fbxo21 regulates the EMT in gastric cancer

**Figure 3:**
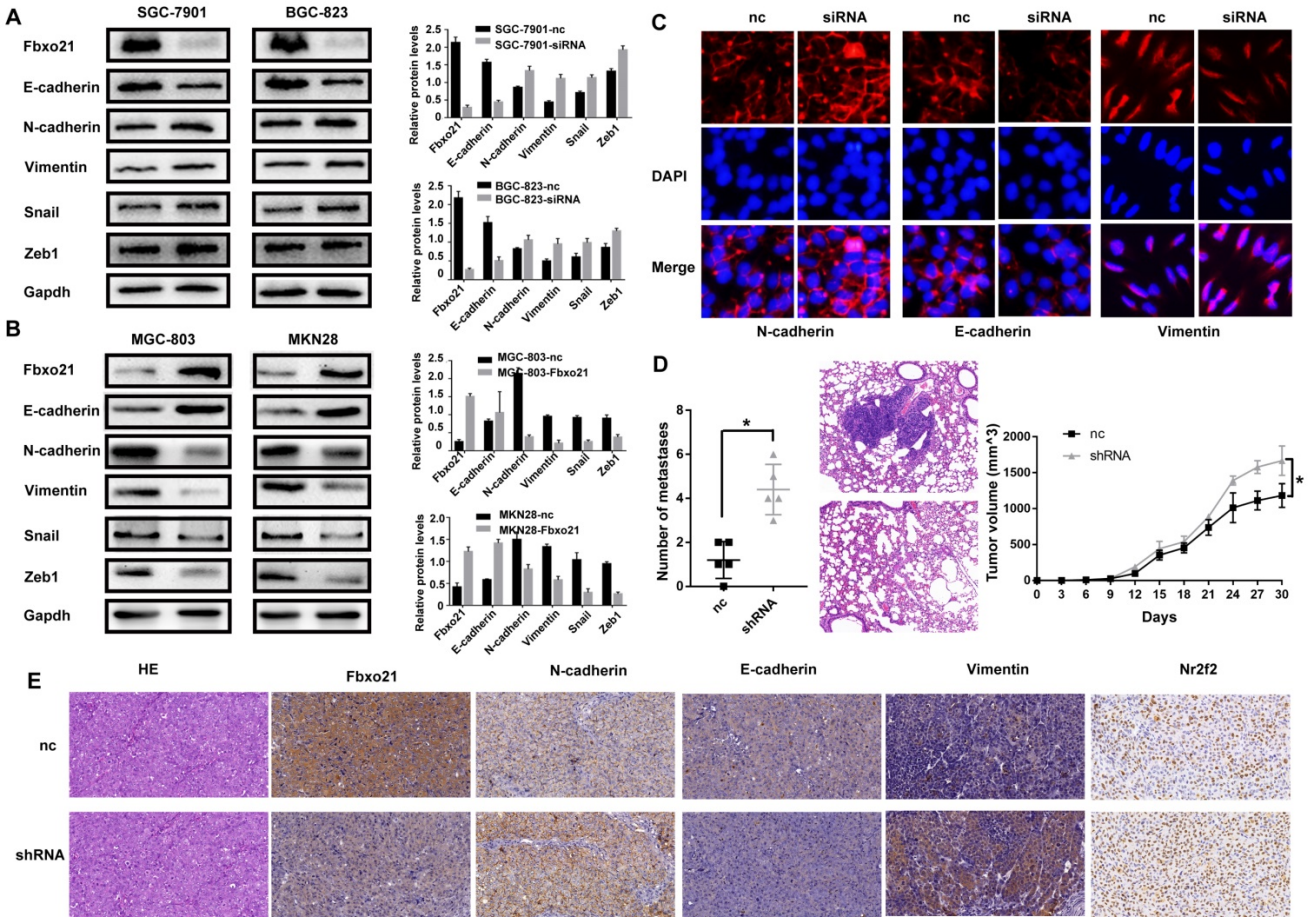
** Fbxo21 plays a crucial role in the EMT in gastric cancer.** To determine whether Fbxo21 suppresses the EMT to inhibit cell migration and invasion, the expression levels of Fbxo21, E-cadherin, N-cadherin, Vimentin, Snail and Zeb1 were detected in all transduced and control cell lines via western blot, immunofluorescent staining and xenograft mouse model. **A.** Fbxo21-silenced SGC-7901 and BGC-823 cells displayed increased expression of N-cadherin, Vimentin, Snail and Zeb1 and decreased expression of E-cadherin. **B.** Fbxo21-overexpressed MGC-803 and MKN28 cells showed increased E-cadherin and decreased N-cadherin, Vimentin, Snail and Zeb1. **C.** Immunofluorescent staining of E-cadherin, N-cadherin and Vimentin in Fbxo-21 silenced SGC-7901 cells. **D.** Fbxo21-silenced SGC-7901 cells had more aggressive lung metastasis ability and growth rate. **E.** Knockdown of Fbxo21 could inhibit EMT *in vivo*.

Considering the important role of EMT process in cell migration and invasion, we wondered if Fbxo21 affected the gastric cancer migration and invasion via the EMT. We studied the protein expression of Fbxo21, E-cadherin, N-cadherin and Vimentin in transduced and non-transduced cells by western blot and immunofluorescent staining. As expected, Fbxo21 knockdown in SGC-7901 and BGC-823 cells down-regulated the epithelial marker E-cadherin, and up-regulated the mesenchymal markers N-cadherin and Vimentin (Figure 3A, 3C). Fbxo21 overexpression in MGC-803 and MKN28 cells increased the levels of E-cadherin and decreased the levels of N-cadherin and Vimentin. In addition, we investigated the upstream transcription factor that regulated EMT. After Fbxo21 knockdown in SGC-7901 and BGC-823 cells, the expression levels of Snail and Zeb1 were significantly increased, while the expressions were decreased in Fbxo21 overexpression MGC-803 and MKN28 cells (Figure 3B). Therefore, we suggested that Fbxo21 inhibited the EMT in gastric cancer by downregulating Snail and Zeb1, two upstream transcription factors of the EMT.

The xenograft mouse model experiments verified the role of Fbxo21 in gastric tumor progression *in vivo*. After knockdown of Fbxo21 expression, the SGC-7901 cells obtained stronger lung metastasis and growth ability (Figure 3D), with up-regulated expression of N-cadherin, Vimentin and down-regulated E-cadherin (Figure 3E).

### 3.5. The Fbxo21 expression level is negatively correlated with the Nr2f2 expression level in gastric cancer

**Figure 4:**
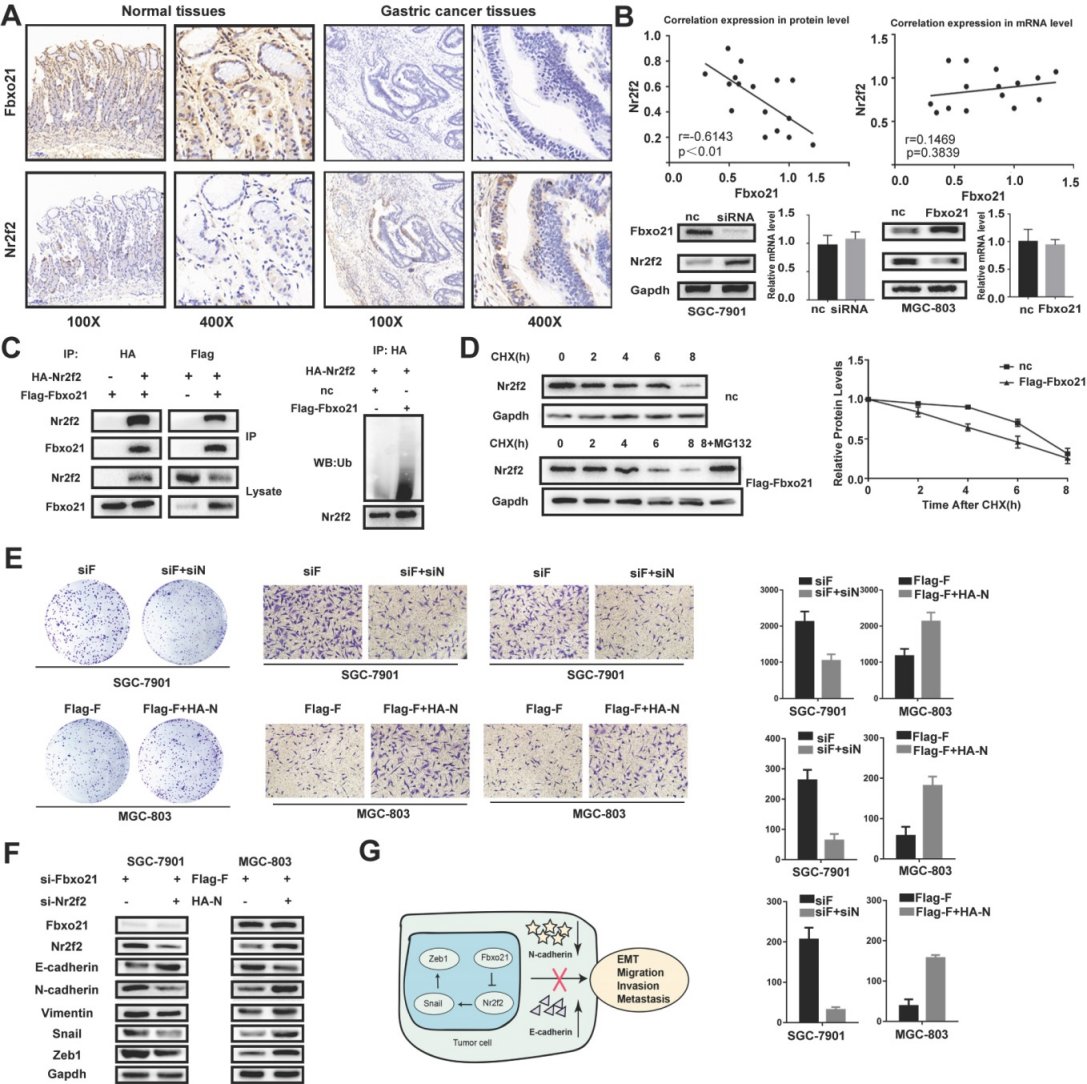
**The relation of Fbxo21 and Nr2f2 in gastric cancer. A.** Immunohistochemical (IHC) staining for Fbxo21 and Nr2f2 in normal tissues and gastric cancer tissues. **B.** Based on Spearman's rank correlation analysis, there was a strong inverse correlation between Fbxo21 and Nr2f2 protein expression in gastric cancer tissues. However, there was no significant correlation between the Fbxo21 and Nr2f2 mRNA levels. The similar results were observed in the gastric cancer cells. **C.** We used co-immunoprecipitation to detect the relationship between Fbxo21 and Nr2f2. Flag-Fbxo21 and HA-Nr2f2 were transfected into MGC-803 cells. Western blotting was performed to detect the specific proteins indicated on the left side of each panel. Nr2f2 ubiquitination was detected by western blotting. Fbxo21 overexpression markedly promoted Nr2f2 ubiquitination. **D.** The protein half-life of Nr2f2 was analyzed following treatment with cycloheximide. Treatment with MG132 (a proteasome inhibitor) inhibited Fbxo21-induced Nr2f2 degradation in MGC-803 cells. Fbxo21-induced growth arrest, migration, invasion and EMT in gastric cancer cells were partially reversed by Nr2f2. **E.** Nr2f2 knockdown decreased the proliferation, migration and invasion of Fbxo21-silenced SGC-7901 cells. Nr2f2 overexpression increased the proliferation, migration and invasion of Fbxo21-overexpression MGC-803 cells. **F.** Nr2f2 knockdown in Fbxo21-silenced SGC-7901 cells increased the expression of E-cadherin and decreased the expression of N-cadherin, Vimentin, Snail and Zeb1. In parallel, Nr2f2 overexpression in Fbxo21-overexpressing MGC-803 cells increased the expression of N-cadherin, Vimentin, Snail and Zeb1 and decreased the expression of E-cadherin. **G.** Schematic diagram of Fbxo21 antitumor function in gastric cancer cells.

Nr2f2 has been reported to play important roles in the occurrence and development of gastric cancer, and identified as one of the potential targets of Fbxo21. We examined the Nr2f2 protein levels in 16 gastric cancer tissues and matched normal tissues via immunohistochemistry. The results showed that the protein expression of Nr2f2 in cancer tissues was significantly higher than that in normal tissues (Figure 4A). In addition, we detected the mRNA levels of Fbxo21 and Nr2f2 in these tissues by RT-PCR. According to Spearman's rank correlation analysis, there was a strong negative correlation between Fbxo21 and Nr2f2 protein levels in gastric cancer tissues. However, we found no significant correlation between Fbxo21 and Nr2f2 mRNA levels (Figure 4B). Furthermore, the Nr2f2 expression significantly increased in Fbxo21-silenced SGC-7901 cells, and reduced in Fbxo21-overexpressed MGC-803 cells.

### 3.6. Fbxo21 induces Nr2f2 ubiquitination and proteasomal degradation

The target specificity of Fbxo21 is determined by recognizing and binding to proteins, which eventually leads to ubiquitination and proteasomal degradation. Nr2f2 is one of the potential targets of Fbxo21 without deep investigation and examination. Thus, we hypothesized that Fbxo21 could combine with Fbxo21, resulting in ubiquitination and proteasomal degradation. We examined the interaction between Fbxo21 and Nr2f2 via reciprocal co-IP of Flag-Fbxo21 and HA-Nr2f2 and detected effect of Fbxo21 overexpression on Nr2f2 ubiquitination level via western blot of ubiquitin (Figure 4C). We also used cycloheximide chase analysis to analyze the dynamic change of Nr2f2 status in MGC-803 cells transfected with Flag-Fbxo21 or the negative control vector over time. We found that the half-life of Nr2f2 in the Fbxo21-overexpressing MGC-803 cells was significantly shorter than that in control cells. In addition, we applied the proteasome inhibitor MG132 to MGC-803 cells transduced with Flag-Fbxo21 (Figure 4D). As expected, the decreased expression of Nr2f2 was blocked in MG132-treated cells. In conclusion, Fbxo21 combines with Nr2f2 and induces its ubiquitination and proteasomal degradation.

### 3.7. Nr2f2 re-expression or inhibition reverses the biological change in Fbxo21-overexpressing or Fbxo21-silenced cell lines

To determine whether Fbxo21 regulates proliferation and the EMT in gastric cancer through the Nr2f2 signaling pathway, we re-expressed or knocked down Nr2f2 in Fbxo21-overexpressing MGC-803 and Fbxo21-silenced SGC-7901 cells. We detected cellular proliferation using colony formation assays, invasion and migration using transwell assays. As expected, inhibition or restoration of Nr2f2 expression in SGC-7901 or MGC-803 cells rescued the effect of exogenous Fbxo21 knockdown or overexpression. The proliferation of Fbxo21-silenced SGC-7901 cells decreased after Nr2f2 inhibition, and the proliferation of Fbxo21-overexpressed MGC-803 cells increased after Nr2f2 re-expression (Figure 4E). Likewise, the migration and invasion of the Fbxo21-silenced SGC-7901 and Fbxo1-overexpressed MGC-803 cells were inhibited or promoted after Nr2f2 inhibition or re-expression. Then, we tested the expression level of the EMT markers. The inhibition of Nr2f2 in Fbxo21-silenced SGC-7901 cells resulted in the partial decrease of Snail, Zeb1, N-cadherin and Vimentin and the partial increase of E-cadherin. Alternatively, the expression levels of these markers showed the opposite trend after the re-expression of Nr2f2 in Fbxo21-overexpressed MGC-803 cells (Figure 4F). In conclusion, these data suggest that Nr2f2 may be a downstream factor of Fbxo21 regulating cell proliferation and the EMT.

## 4. Discussion

This study investigated the biological role of Fbxo21 and its mechanisms in gastric cancer. The data showed that Fbxo21 expression was reduced in gastric cancer tissues and correlated with poor prognosis of gastric cancer patients. Moreover, reduced Fbxo21 expression promoted cancer cell proliferation, migration and invasion, especially the EMT partially through Nr2f2 signaling pathway. These findings indicated the key role of Fbxo21 as a tumor suppressor and revealed a new molecular mechanism underling the development and progression of gastric cancer.

Fbxo21, a novel-estimated member of F-box family proteins, functions as a subunit of E3 ubiquitin ligase in proteasomal process. Fbxo21 was reported to participate in the development of a few human malignances 18, 19, and the highest mutation frequency in gastric cancer is 4.17% (https://www.phosphosite.org). We detected Fbxo21 expression in gastric cancer in MERAV website and found that Fbxo21 expression was much lower in gastric cancer tissues than normal mucosa. Then we examined the Fbxo21 expression via western blot and immunohistochemistry in specimens from gastric cancer patients. The results were consistent with mRNA expression data from MERAV. Moreover, the data from TCGA database showed that the decrease of Fbxo21 expression in gastric cancer patients was related to the reduction of 5-year survival rate. Therefore, we have confirmed that the decreased expression of Fbxo21 indicates the poor prognosis of human gastric cancer.

Then we examined the effect of different Fbxo21 expression levels on the biological function of gastric cancer cells *in vivo* and *in vitro*. We used CCK8 and colony formation assays to detect the cell proliferation, and transwell assays for migration and invasion. Our results indicated that overexpression of Fbxo21 inhibited the proliferation of MGC-803 and MKN28 cells, but the knockdown of Fbxo21 stimulated the proliferation of SGC-7901 and BGC-823 cells. Moreover, we found that increasing Fbxo21 expression inhibited the migration and invasion of MGC-803 and MKN28 cells, while reducing Fbxo21 expression had the opposite effect on SGC-7901 and BGC-823 cells. We also detected the role of Fbxo21 in the EMT of gastric cancer. The EMT is a process of cadherin conversion that is generally considered to enhance the motility and invasiveness of cancer cells, which is critical to tumor metastasis 5. We examined the representative proteins of EMT, such as E-cadherin, N-cadherin and Vimentin. The results indicated that Fbxo21 status regulated the EMT phenotype of gastric cancer. Taken together, Fbxo21 functions as a tumor suppressor in gastric cancer.

As a new-found and estimated F-box family protein member, Fbxo21 showed its function mainly in immunology, such as EBV infection 16. Only a few studies had shown that Fbxo21 promoted the degradation of some oncoproteins, such as EID1 and P-gp 17-19, via ubiquitination and subsequent proteasomal degradation. Fbxo21 may regulate cellular growth and invasiveness in this manner, and we would like to find a specific target of Fbxo21 in gastric cancer. As shown in previous research, Nr2f2 was one of the candidate targets of Fbxo21 based on the MS analysis without deep study and confirmation 9, and played an important role in the EMT in many cancers through Snail, Smad4, Smad7 and other translational factors 25, 26, 28. Considering the important role of Nr2f2 in the EMT process revealed by these studies, we supposed that the Fbxo21 may regulate gastric cancer biological process via Nr2f2. We determined the role of Nr2f2 in gastric cancer and the relation between Fbxo21 and Nr2f2. In accordance with previous studies, the expression of Nr2f2 in 16 gastric cancer tissues was significantly higher than in the normal tissues. In addition, Fbxo21 protein expression was inversely associated with Nr2f2 protein expression in the gastric cancer tissues, but not the mRNA level. The knockdown of Fbxo21 resulted in an accumulation of Nr2f2. In contrast, the overexpression of Fbxo21 significantly downregulated the Nr2f2 levels. Fbxo21 was reported to combine with CUL1 and SKP1 to form a SCF complex, which mediated the downstream protein ubiquitination and degradation 17. In order to reveal the underlying mechanism and interaction between Fbxo21 and Nr2f2, we did co-immunoprecipitation and western blot. The results indicated that Fbxo21 could bind to Nr2f2 and promote the ubiquitination and proteasomal degradation of Nr2f2, thus reducing the half-life of Nr2f2. MG132 treatment could block the down-regulation of Nr2f2 by Fbxo21. Thus, we confirmed that Nr2f2 was the specific ubiquitylation target of Fbxo21 in gastric cancer. As reported in the literature, phosphorylation of the F-box recognition motif on the substrate is necessary for an efficient substrate-ligase interaction 10. We could use proteomic data to find the phospho-degron in Nr2f2 and the exact binding site of these two proteins in future studies. To the end, we re-expressed Nr2f2 in the Fbxo21-overexpressed MGC-803 cells, and found the transduction of HA-Nr2f2 increased the cell proliferation, migration and invasion. We also knocked down Nr2f2 expression in the Fbxo21-silenced SGC-7901 cells, and found the decreased expression of Nr2f2 reversed the effect of Fbxo21 down-regulation. Moreover, the EMT phenotype was also reversed by Nr2f2 re-expression or silence. Some upstream transcription factors of the EMT were also detected via western blot. We found that Snail and Zeb1 showed significant variation in these experiments. Therefore, the EMT could be stimulated by reduced Fbxo21 expression, via Nr2f2/Snail signaling.

In conclusion, we confirmed that Fbxo21 played a crucial role in tumor growth, metastasis and prognosis via Nr2f2/Snail pathway (Figure 4G). The status of Fbxo21 gene or protein would likely be a potential biomarker for clinical prognosis and an individualized clinical therapy target in gastric cancer.
